# Characteristics and phylogenetic analysis of the complete chloroplast genome of *Thalictrum przewalskii* Maxim. 1877

**DOI:** 10.1080/23802359.2026.2699491

**Published:** 2026-08-13

**Authors:** Qinyi Wang, Shihao Jiang, Xingyu Du, Yanru Zhang, Yi Tang, Jiazhen Wu, Yatong Zeng, Liang Pang, Xiuhua Wang, Zhixi Fu, Jieli Yue

**Affiliations:** ^a^ Key Laboratory of Land Resources Evaluation and Monitoring in Southwest (Sichuan Normal University), Ministry of Education, Chengdu, China; ^b^ Sichuan Tianshengyuan Environmental Services Co., Ltd, Chengdu, China; ^c^ Xizang Shengyuan Environmental Engineering Co., Ltd, Lhasa, China; ^d^ Beijing Dongfangdecai School, Beijing, China; ^e^ College of Life Sciences, Sichuan Normal University, Chengdu, China; ^f^ Sichuan Guoce Testing Technology Co., Ltd, Chengdu, China; ^g^ Meishan Eco-environmental Monitoring Center Station of Sichuan Province, Meishan, China

**Keywords:** Plastome, coding sequences, *thalictrum*, phylogenetic relationship

## Abstract

This study first reported the complete chloroplast genome of *Thalictrum przewalskii* Maxim. and analyzed its structure and phylogeny. This circular double-stranded genome is 155,922 bp long with a GC content of 38.4%, containing an 85,347 bp LSC region, a 17,613 bp SSC region, and two 26,481 bp IR regions. A total of 131 functional genes were annotated. Phylogenetic analysis of 21 species indicated that* Thalictrum przewalskii* Maxim. is most closely related to* Thalictrum baicalense* Turcz. ex Ledeb., defining its phylogenetic position and providing a molecular basis for future studies on *Thalictrum* species.

## Introduction

The genus *Thalictrum* is an important genus with significant phylogenetic and economic value in Ranunculaceae. There are about 200 species of this genus worldwide, mainly distributed in the Northern Temperate Zone (Ling et al. 2024). There are more than 70 species and varieties of *Thalictrum* in China, mainly distributed in the mountainous areas of Southwest, Northwest and North China (Chen et al. 2024). At present, many plants of this genus have been widely used as folk medicines for the treatment of fever, inflammation, jaundice, dysentery, rheumatoid arthritis and other diseases (Walter et al. 2020). *Thalictrum przewals*kii Maxim. 1877 is mainly distributed in Gansu, Qinghai, Sichuan, Shaanxi, Shanxi, Hebei and other places, growing in mountain forests, shrub edges and grass slopes at an altitude of 1800–4000 meters (Zeng et al. 2025).

Compared with nuclear genome and mitochondrial genome, chloroplast genome has conservative and compact structure and moderate nucleotide substitution rate, making it an ideal molecular carrier for plant phylogenetic analysis (Jansen et al. 2007; Daniell et al. 2016). Most angiosperm chloroplast genomes have a typical quadripartite circular structure, consisting of a pair of inverted repeats (IRs), a large single-copy (LSC) region, and a small single-copy (SSC) region (Cauz-Santos 2025).

In this study, we sequenced, assembled, annotated and analyzed the characteristics of the complete chloroplast genome of *Thalictrum przewalskii*, and explored its phylogenetic relationship within the genus *Thalictrum* by combining data of closely related species. The results of this study can provide basic data support for the phylogeny of *Thalictrum* plants (Gu et al. 2020; Yan et al. 2023; Gu et al. 2024).

## Materials and methods

### Plant material

**Figure 1.**
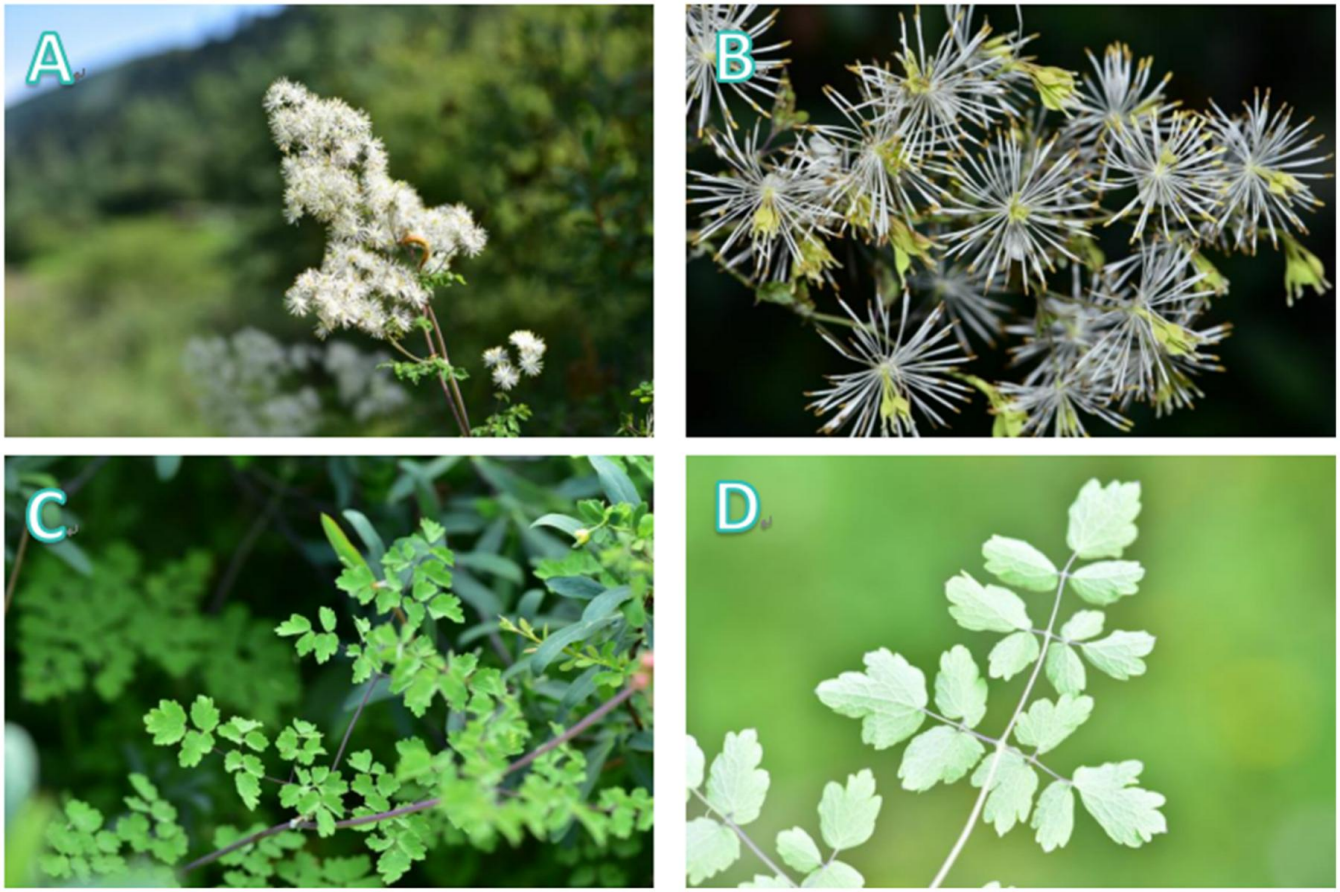
Photos of *Thalictrum przewalskii* taken by Dechang Meng, with no copyright issues. Panel a shows the panicle in the distance; Panel B shows the panicle in close view; Panel C shows the leaves in the distance; Panel D shows the abaxial surface of the leaves in close view. This plant is a perennial herb. The stem is 50–120 cm tall and glabrous. The lower cauline leaves are biternately compound with long petioles; the leaflets are 0.9–3.5 cm wide, with short pubescence or glabrous on the abaxial surface. The panicle has numerous flowers; the sepals are white or pale yellowish-green; the upper part of the filaments is linear-oblanceolate.

*Thalictrum przewalskii* was collected in the wild in Seda County, Garzê Tibetan Autonomous Prefecture, Sichuan Province, China (N 31.750°, E 100.476°) (Figure 1). After identification by Dr. Zhixi Fu, these materials have been deposited in the Herbarium of Sichuan Normal University, China (SCNU) (https://bio.sicnu.edu.cn/; contact person: Dr. Zhixi Fu, email: fuzx2017@sicnu.edu.cn). The voucher specimen number is SDX0216. After collecting the species, fresh leaves were stored in sealed bags with silica gel desiccant.

### DNA extraction and sequencing

Total genomic DNA was extracted from fresh leaves using the CTAB DNA extraction protocol (Allen et al. 2006). DNA library was constructed using Illumina PairedEnd DNA Library Kit (Illumina Inc. San Diego, CA, USA). Sequencing of qualified libraries was performed on the Illumina NovaSeq 6000 platform (NovoGene Inc., Beijing, China) with a read length of 150 bp. Finally, raw sequences were obtained using the Illumina Genome Analyzer through the sequencer of Illumina Inc. (San Diego, CA, USA). This study used BWA (version v0.7.17) to generate depth of coverage (Figure S1) (minimal depth = 509 ×; maximal depth = 7711 ×; average depth = 3313.31 ×) (https://www.protocols.io/view/generating-sequencing-depth-and-coverage-map-for-o-4r3l27jkxg1y/ v1).

### Genome assembly and annotation

In this study, SPAdes v3.15.1-Linux software was used for data assembly with default parameters (Bankevich et al. 2012). To evaluate the assembly quality, circular maps were identified by Bandage software (Wick et al. 2015). Subsequently, PGA (Qu et al. 2019) was used to annotate the chloroplast genome sequence, and the results were verified by Geneious R11 (Kearse et al. 2012). The annotation results were visualized using the CPGview program (Figures S2 and S3) (Liu et al. 2023).

**Figure 2.**
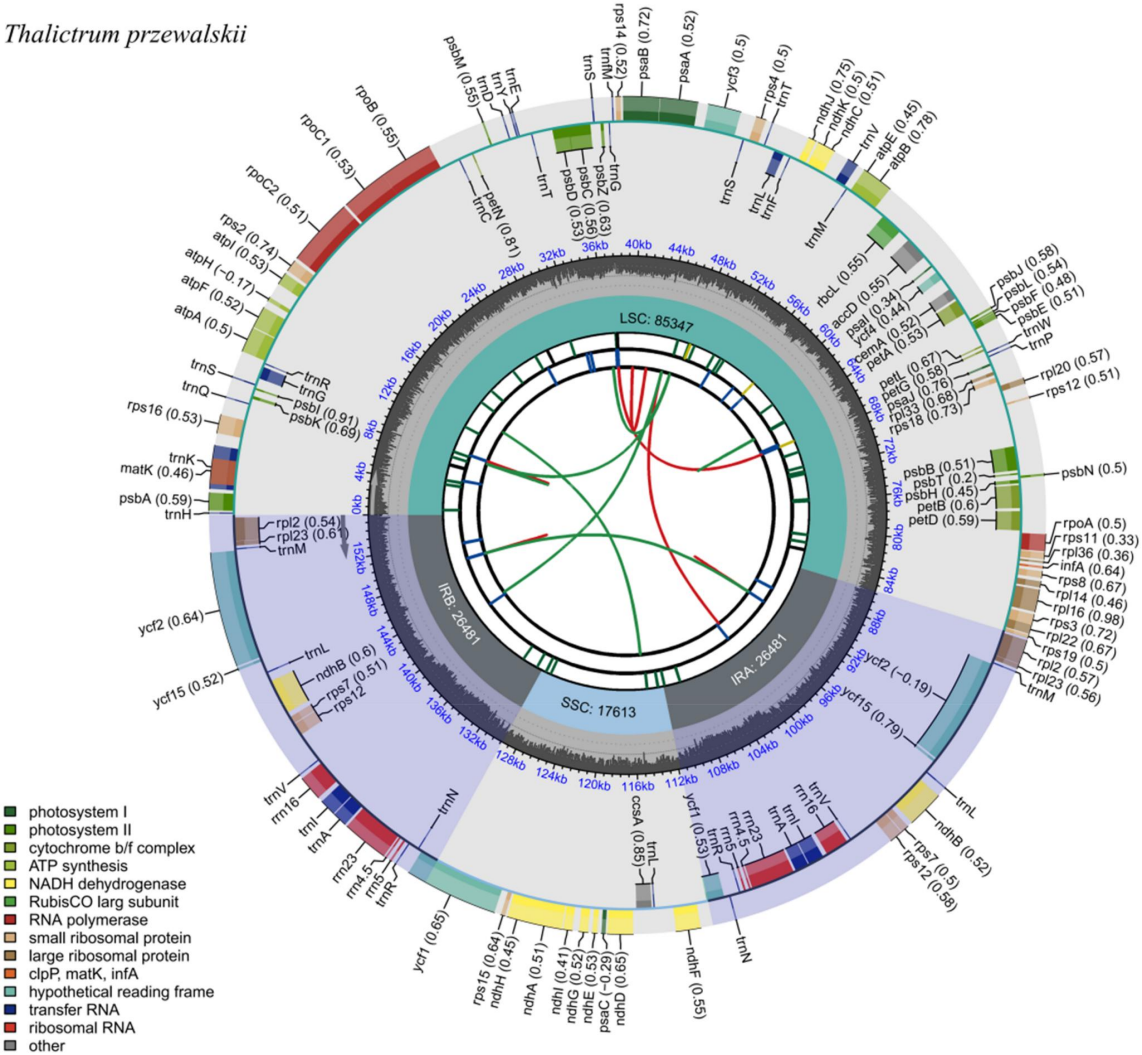
Circular gene map of the complete chloroplast genome of *Thalictrum przewalskii*.The map shows a typical quadripartite structure, consisting of six independent concentric tracks from the center outward. The innermost track displays dispersed repeat sequences in the genome, where red arcs denote forward repeats and green arcs denote reverse repeats, visually presenting the distribution and type characteristics of repetitive elements. The second track highlights long tandem repeats in the genome with blue bars, clearly marking the specific positions and abundance of long repetitive sequences. The third track marks short tandem repeats (microsatellites) with green bars, clearly illustrating the distribution pattern of microsatellite sequences across the genome. The fourth track annotates the four major functional regions of the chloroplast genome, namely the large single-copy region (LSC), the small single-copy region (SSC), and a pair of inverted repeat regions (IRA/IRB), explicitly defining the structural partitioning of the genome. The fifth track presents the GC content distribution of the entire chloroplast genome, with variations in bar depth reflecting differences in GC content across different genomic regions. The outermost track annotates all genes with colored boxes, which are color-coded by functional group. Genes with boxes inside the circle are transcribed in the clockwise direction, while those with boxes outside the circle are transcribed in the counterclockwise direction. The numbers in parentheses following gene names represent the codon usage bias of the corresponding genes.

**Figure 3.**
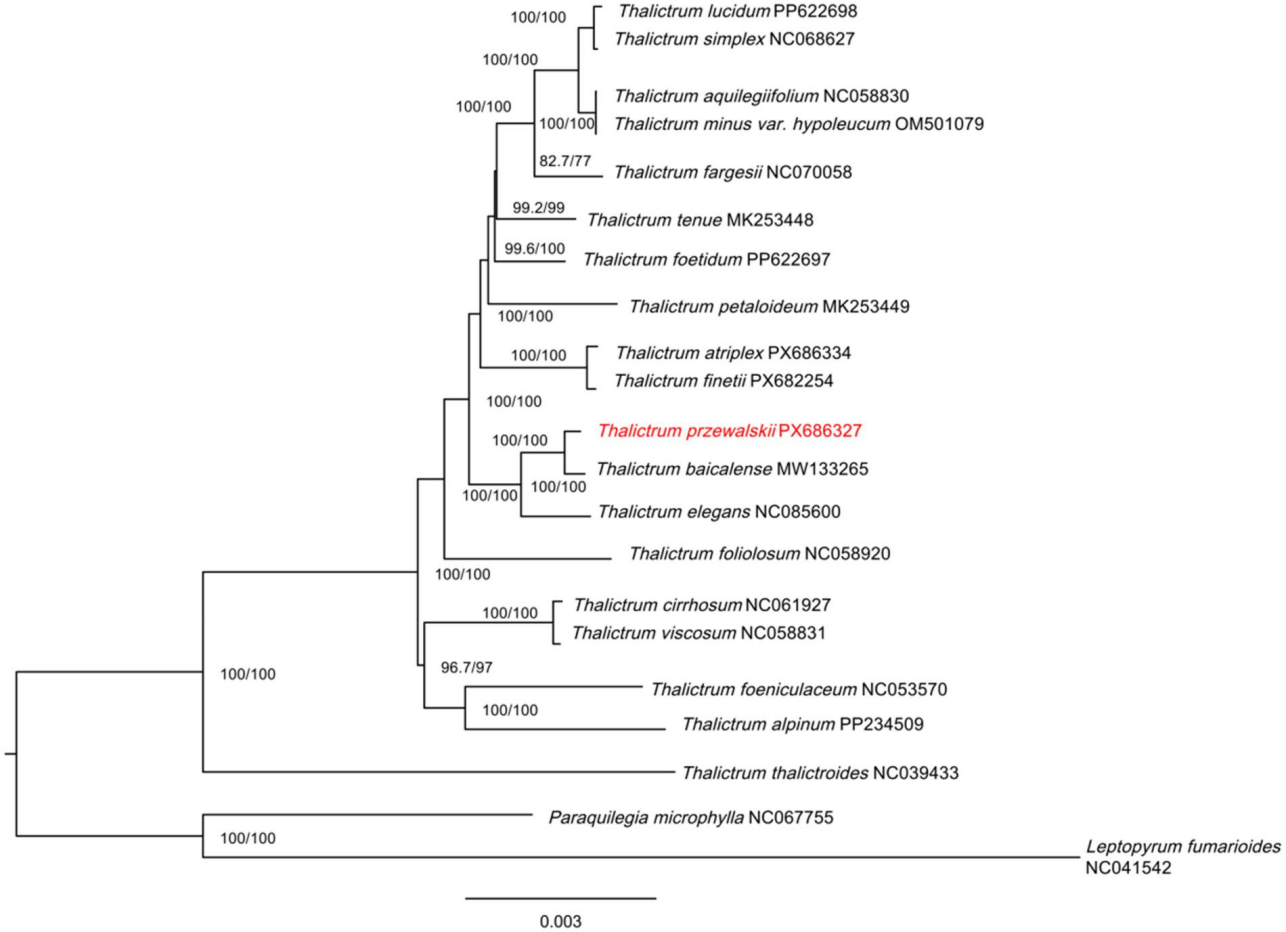
The best maximum likelihood (ML) phylogenetic tree inferred from 21 chloroplast genomes (bootstrap values are labeled on the branches). Species marked in red were newly sequenced in this study. The sequences used include: *Thalictrum simplex* NC068627 (Xiang et al. 2023), *Thalictrum aquilegiifolium* NC058830 (Xiang et al. 2023), *Thalictrum minus* var. *hypoleucum* OM501079 (Xiang et al. 2023), *Thalictrum fargesii* NC070058 (Pu et al. 2022), *Thalictrum atriplex* PX686334 (Zeng et al. 2022), *Thalictrum finetii* PX682254 (Xiang et al. 2023), *Thalictrum viscosum* NC058831 (Cai et al. 2022), *Thalictrum thalictroides* NC039433 (unpublished), *Thalictrum baicalense* MW133265 (He et al. 2021), *Thalictrum przewalskii* PX686327 (the sequenced species), *Thalictrum lucidum* PP622698 (Xiang et al. 2026), *Thalictrum foetidum* PP622697 (Cai-Feng et al. 2019), *Thalictrum alpinum* PP234509 (unpublished), *Thalictrum elegans* NC085600 (Cheng et al. 2024), *Thalictrum cirrhosum* NC061927 (Cai et al. 2022), *Thalictrum foliolosum* NC058920 (Pu et al. 2022), *Thalictrum foeniculaceum* NC053570 (Lin et al. 2021), *Thalictrum petaloideum* MK253449 (Chae et al. 2025), *Thalictrum tenue* MK253448 (Xu et al. 2020), *Paraquilegia microphylla* NC067755 (unpublished), *Leptopyrum fumarioides* NC041542 (unpublished).

### Phylogenetic analysis

In this study, 19 chloroplast genome sequences of *Thalictrum* and 2 closely related genera were downloaded from the GenBank database, then imported into MAFFT v7.526 software for multiple sequence alignment (Katoh and Standley 2013), and then the GTRGAMMA model (Stamatakis 2014) was used to construct the ML phylogenetic tree (Miller et al. 2010) with 1000 bootstrap replicates.

## Results

### Genome structure analysis

The complete chloroplast genome sequence of *Thalictrum przewalskii* is 155,922 bp in length, with a typical quadripartite structure observed in most terrestrial plants (Figure 2) (Cauz-Santos 2025). A pair of 26,481 bp IR regions (IRa and IRb) separate the 85,347 bp LSC region and 17,613 bp SSC region in the chloroplast genome. The GC content of the chloroplast genome of *Thalictrum przewalskii* is 38.4%. A total of 131 genes were annotated (111 unique genes) (Figure 2, Table S1), including 86 protein-coding genes, 37 tRNA genes and 8 rRNA genes. In addition, the genome contains 16 cis-splicing genes, including 15 genes with one intron (*ndhB*, *ndhA*, *petB*, *petD*, *atpF*, *rpl16*, *rpl2*, *rps16*, *rpoC1*, *trnK-UUU*, *trnG-UCC*, *trnL-UAA*, *trnV-UAC*, *trnI-GAU*, and *trnA-UGC*) and one gene with two introns (*ycf3*) (Figure S2). The gene rps12 is a typical trans-spliced gene (Figure S3), whose 5′ exon is located in the large single-copy region (LSC), and the 3′ exon is located in the inverted repeat region (IR). The complete genome sequence has been deposited in GenBank with the accession number PX686327.

### Phylogenetic analysis

In this study, 19 complete plastid genome sequences of *Thalictrum* species and 2 outgroup plastid genomes (*Paraquilegia microphylla* (Royle) Drumm. & Hutch. and *Leptopyrum fumarioides* (L.) Rchb.) were used for phylogenetic analysis. The results showed that *Thalictrum przewalskii* and *Thalictrum baicalense* formed a sister group (support rate 100/100), and the clade formed by them formed a sister group with *Thalictrum elegans* (support rate 100/100). *Paraquilegia microphylla* and *Leptopyrum fumarioides* formed a sister group (Figure 3).

## Discussion and conclusions

This study first presents the complete chloroplast genome of *Thalictrum przewalskii*, which exhibits a typical angiosperm circular quadripartite structure with LSC, SSC and paired IR regions (Cauz-Santos 2025). Compared with published congeneric plastomes, the 155,922 bp chloroplast genome of *T. przewalskii* fits the genus size range (154,000–157,000 bp) without obvious expansion or shrinkage, with its 38.4% GC content matching congeners including *T. baicalense* and *T. elegans* (both 38.4%). Structurally, its LSC, SSC and each IR span 85,347 bp, 17,613 bp and 26,481 bp respectively, nearly identical to *T. baicalense* (85,258 bp;17,637 bp;26,482 bp) and *T. elegans* (85,265 bp;17,609 bp;26,480 bp). Annotated 131 functional genes (86 protein-coding, 37 tRNA, 8 rRNA) are consistent with other *Thalictrum* species. These data confirm high chloroplast genomic conservation across the genus (Zhao et al. 2020; Wu et al. 2021).

Phylogenetic analyses of *Thalictrum* and related genera based on chloroplast genomes revealed that *Thalictrum* is a strongly supported monophyletic group (100/100), sister to *Paraquilegia* and *Leptopyrum*. These results agree with previous studies (Xiang et al. 2023), confirming the phylogenetic position of *Thalictrum* in Ranunculaceae.This Chinese endemic grows on the eastern Qinghai-Tibet Plateau, providing clues for regional biodiversity research (Zeng et al. 2025). Most nodes get high bootstrap support, yet several clades have modest values (e.g. 82.7/77), implying incomplete lineage sorting or ancient hybridization. Owing to uniparental inheritance and absent recombination, chloroplast data alone cannot resolve intricate evolutionary histories. Future work should combine nuclear SNPs and low-copy genes for independent evidence to separate sorting from hybridization and build more reliable *Thalictrum* phylogeny.

In summary, this study reports for the first time the complete chloroplast genome of *Thalictrum przewalskii*. The genome exhibits a typical quadripartite structure with a total length of 155,922 bp, a GC content of 38.4%, and contains 131 functional genes. Phylogenetic analysis robustly places *T. przewalskii* as a sister species to *T. baicalense*, clarifying its phylogenetic position within the genus *Thalictrum*. These findings provide valuable molecular resources for species identification, future phylogenetic studies, and conservation efforts of *Thalictrum* species, particularly those endemic to the Qinghai-Tibet Plateau and adjacent regions.

## Supplementary Material

Supplemental Material

Supplemental Material

Supplemental Material

## Data Availability

The genome sequence data that support the findings of this study are openly available in GenBank of NCBI at (https://www.ncbi.nlm.nih.gov/) under accession no. PX686327.The associated BioProject, SRA, and BioSample numbers are PRJNA1455421, SRR38219066, and SAMN57383929, respectively.
